# Patisiran exposure in early pregnancy: a case report

**DOI:** 10.1177/17562864241239755

**Published:** 2024-03-26

**Authors:** Valentin Loser, Thomas Baumgartner, Hélène Legardeur, Alice Panchaud, Marie Théaudin

**Affiliations:** Service of Neurology, Department of Clinical Neurosciences, Lausanne University Hospital, University of Lausanne, Rue du Bugnon 44, Lausanne 1011, Switzerland; Service of Neurology, Department of Clinical Neurosciences, Lausanne University Hospital, University of Lausanne, Lausanne, Switzerland; Woman–Mother–Child Department, Lausanne University Hospital, University of Lausanne, Lausanne, Switzerland; Service of Pharmacy, Lausanne University Hospital, University of Lausanne, Lausanne, Switzerland; Institute of Primary Health Care, University of Bern, Bern, Switzerland; Service of Neurology, Department of Clinical Neurosciences, Lausanne University Hospital, University of Lausanne, Lausanne, Switzerland

**Keywords:** amyloidosis, case report, hATTR, Patisiran, pregnancy, siRNA

## Abstract

We describe here the first case of exposure to patisiran treatment, a small interfering RNA molecule, during early pregnancy of a 36-year-old woman with symptomatic hereditary transthyretin-related amyloidosis. There were no major complications during pregnancy and delivery, except for a postpartum hemorrhage due to uterine atony. Vitamin A levels had to be closely monitored during pregnancy, and vitamin A substitution adapted accordingly. There was no sign of minor or major congenital abnormalities of the baby. One month after delivery, the patient showed slight clinical and electrophysiological signs of neuropathy progression due to patisiran treatment withdrawal. Patisiran infusions were resumed 3 months after delivery. Due to the unknown teratogenic potential of patisiran, the risk of neuropathy worsening associated with withholding treatment must of course be weighed against a potential teratogenic risk of treatment during pregnancy. Vitamin A levels need to be closely assessed, and substitution must be adapted accordingly, to avoid embryofetal adverse outcome due to vitamin A deficiency or toxicity.

## Case

This 36-year-old woman was diagnosed with symptomatic hereditary transthyretin-related (hATTR) amyloidosis due to a *p.Val50Met TTR* mutation in 2017. Her father died of hATTR amyloidosis at the age of 38 years old and two siblings also developed the disease. Her first symptoms started in 2017, at the age of 30, with a progressive lower limb small fiber neuropathy and mild autonomic symptoms (orthostatic hypotension, dysuria, and constipation). She also complained of occasional palpitations, but extensive cardiac workup, including cardiac MRI, was within normal range. RNA interference treatment with patisiran, 300 µg/kg IV every 3 weeks, was introduced at the end of 2019. On treatment, neurological symptoms remained stable, as evidenced by functional and severity scores, electrochemical skin conductance (ESC), and nerve conduction studies (NCS). To prevent deficiency related to the drug’s mode of action, vitamin A substitution with 4000 IU three times a week was introduced at patisiran onset.

In December 2022, the patient informed us of an unplanned pregnancy (she already had three healthy children). Despite information on the uncertainties on the risks for the pregnancy due to patisiran treatment,^1^ low levels of prealbumin and vitamin A, the patient and her husband decided to continue with the pregnancy. She thus received the last dose of patisiran during the third week of amenorrhea (last dose given before the patient informed us of the pregnancy), and treatment was then discontinued. A close follow-up with a maternofetal medicine specialist was planned, including monthly dosage of serum vitamin A and prealbumin, and fetal ultrasound follow-up at 13, 17, 22, 31, and 34 weeks of gestation. Vitamin A substitution was increased at 4000 IU per day at the beginning of pregnancy given the uncertainty on available circulating vitamin A (low levels of serum vitamin A) (Table 1). Substitution was discontinued at 3 months of gestation, when vitamin A levels were within normal ranges. There was a progressive increase in prealbumin (TTR) levels over months (Table 1). The pregnancy was harmonious with a good fetal growth. No fetal malformations were detected. After a spontaneous labor, the patient delivered a healthy male infant at 40 weeks of gestation with an APGAR score at 9/9/9, birth weight 3540 g, length 49 cm, and a normal cranial perimeter at 34 cm. His clinical exam at day 2 was normal, including his neurological exam. Delivery was complicated with a postpartum hemorrhage of 1400 mL, due to uterine atony, treated with sulprostone then a Bakri balloon and tranexamic acid perfusions. A postpartum anemia at 84 g/L was treated with iron infusion. There was no sign of minor or major congenital abnormalities. The patient decided to breastfeed her baby until resuming of patisiran. Follow-up of the child’s psychomotor development was then taken over by the primary care pediatrician, with check-ups scheduled in accordance with the recommendations of the Swiss Society of Pediatrics (i.e. check-ups at 1, 2, 4, 6, and 9 months, then at 1, 1.5, 2, 3, 4, 6, 10, and 14 years).

**Table 1.** Serum vitamin A and prealbumin (TTR) levels during pregnancy.

Follow-ups	1 month	1.5 months	2 months	3 months	4 months	6 months
Vitamin A (µmol/L)	**0.2**	**0.4**		1.3	1.6	1.5
Prealbumin (g/L)	**<0.03**	**0.04**	**0.09**	**0.14**	**0.17**	**0.18**

Vitamin A normal value 1.05–2.09 µmol/L, prealbumin normal value 0.2–0.4 g/L.

TTR, transthyretin-related.

Abnormal values are marked in bold.

During pregnancy, the patient complained of a slight accentuation of a preexisting lower limbs loss of sensitivity and a slight increase in the frequency of orthostatic dizziness, without any syncope. Constipation and dysuria remained unchanged. At last neurological follow-up performed 1 month after delivery, clinical examination demonstrated pain hypoesthesia in both fingertips and feet, with no other abnormalities. Functional and severity scores were globally stable compared with the last follow-up 9 months before. Screening of orthostatic hypotension with Schellong test was negative. ESC measured with Sudoscan^®^ (Impeto Medical, Paris, France) showed normal skin conductance in hands and feet, with similar values compared to the previous follow-up. NCS showed a slight reduction in motor and sensory sum scores compared to the last follow-up. Functional and severity scores, ESC, and NCS parameters from the pre- and post-pregnancy follow-ups are displayed in Table 2. Clinical and NCS scores are further described in the Appendix. Patisiran infusions were resumed 3 months after delivery.

**Table 2.** Functional and severity scores, ESC, and NCS parameters pre- and post-pregnancy.

Follow-ups	Norfolk QOL-DN	SFN-SIQ	CADT	R-ODS	PND	NIS	ESC (hands/feet) (μS)	NCS motor sum score	NCS sensory sum score
Before pregnancy	3	12	13	48	1	3	66/71	20.5	26
After pregnancy	6	12	12	48	1	4	66/73	17.3	20.8

CADT, Compound Autonomic Dysfunction Test; ESC, electrochemical skin conductance; NCS, nerve conduction study; NIS, Neuropathy Impairment Score; PND, Polyneuropathy Disability Score; QOL-DN, quality of life-diabetic neuropathy; R-ODS, Rasch-built Overall Disability Score; SFN-SIQ, small fiber Neuropathy-Symptom Inventory Questionnaire.

## Discussion

We present here the first case of exposure to patisiran in early pregnancy. As for most pharmacological treatments, pregnant women have been excluded from clinical studies assessing patisiran effectiveness and safety. In the APOLLO and in the global open label extension studies, women of childbearing age must have a negative pregnancy test and use two highly effective methods of contraception before inclusion.^2,3^ A patisiran pregnancy exposure registry is currently ongoing (ALN-TTR02-010, NCT05040373), sponsored by Alnylam Pharmaceuticals. Women exposed to patisiran treatment at any point of the pregnancy can be enrolled in the registry, with contact information listed on the Food and Drug Administration website.^4^

In this case report, the exposure to patisiran has been limited to early pregnancy but without a clear definition on up to when exactly. While the last injection took place during the third week after the last menstruation period, pharmacokinetic studies show a very flat steady-state concentration curve suggesting that exposure may have persisted beyond the classical seven times the 3-day half-life of patisiran (usually, a full drug wash-out is expected after seven half-lives). This is the reason why the potential impact of patisiran treatment during organogenesis was raised by the interprofessional team specialized in the management of pregnancies complicated by chronic neurological diseases, who thus closely monitored this high-risk pregnancy.

Currently, there are no reported human exposures to patisiran during pregnancy. Animal data suggest no risk at human therapeutic doses, but not at toxic doses to humans in rodents and rabbits.^5^ The high molecular weight of patisiran (i.e. 14,304 daltons) and the properties of its lipid nanoparticles coating (large size, polyethylene glycol content) make placental transfer unlikely, unless it has a specific transfer mechanism.^6,7^ The lipid nanoparticle coating, the same as the one used in mRNA CoVID-19 vaccines, is known to induce some level of systemic inflammation.^8^ It is unknown whether this immunogenicity may induce pregnancy or neonatal adverse events. However, women vaccinated during pregnancy with mRNA CoVID-19 vaccine did not experience higher adverse pregnancy or neonatal outcomes in recent studies, suggesting a reasonable safety profile of lipid nanoparticles coating.^9,10^ In the absence of human reproductive safety information, the Swiss summary of product characteristics states that pregnancy should be excluded before starting treatment with patisiran, and women of childbearing age should use effective contraception. In women planning a pregnancy, patisiran and vitamin A supplementation should be discontinued, and serum vitamin A levels should be monitored and been returned to normal before conception is attempted. The child’s psychomotor development should then be monitored according to local pediatric recommendations.

Vitamin A imbalances linked to the mechanism of action of patisiran itself, or to the need for vitamin A substitution during its use, could affect the outcome of pregnancy. TTR protein, targeted by patisiran, is directly involved in the transport of vitamin A (retinol) in association with retinol-binding protein. Following patisiran treatment, serum vitamin A levels decreases, with a mean reduction over 18 months of 62.4%.^11^ Therefore, to avoid ocular toxicity due to a vitamin A deficiency (VAD), a substitution with vitamin A is mandatory, at a dose of 2500 IU/day according to Swiss recommendations. Vitamin A is a crucial micronutrient for the fetus and exerts systemic effects on several fetal organs and skeleton. VAD, particularly in the third trimester of pregnancy, has been shown to be associated with an increased risk of preterm delivery, maternal anemia and infections, low birth weight, and overall newborn mortality and morbidity.^12,13^ On the other hand, high vitamin A intake, especially in the first trimester of pregnancy, can be teratogenic.^14^ Therefore, in pregnant women exposed to patisiran, serum vitamin A levels must be closely monitored, and vitamin A substitution adapted accordingly. According to the World Health Organization, vitamin A substitution of pregnant women should not exceed 10,000 IU daily or 25,000 IU weekly, to avoid teratogenicity.^15^ In our patient, vitamin A levels remained relatively low during the first trimester of pregnancy, before being corrected by a more intensive vitamin substitution.

The patient’s delivery was complicated by postpartum hemorrhage due to uterine atony. There seems to be no causal relationship between this complication and patisiran. Indeed, patisiran treatment is not associated with hemorrhagic complications or the occurrence of coagulation disorders, and VAD seems not to be associated with an increased risk of uterine atony.^16^

The uncertainties on the risks for the pregnancy need to be weighed against the risk of neuropathy worsening associated with withholding treatment during the pregnancy, pending further studies. In the literature, untreated hATTR patients have shown variable rates of neuropathy progression. In the Conceição *et al.*^17^ study, most of untreated hATTR patients with a *p.Val50Met* mutation showed signs of clinical and electrophysiological neuropathy worsening during follow-ups, as early as 12 months. In our patient, stopping patisiran treatment during pregnancy and breastfeeding (11 months in total), was probably associated with a slight progression of the neuropathy, as evidenced by an accentuation of pre-existing sensory and autonomic symptoms and a mild decrease in amplitude of lower limbs motor and sensory nerve action potential (SNAP) in NCS, with however stable ESC and functional and disease severity clinical scores. Yet, those variations in NCS values could be related to an interexaminer variability and do not reach the threshold of a significant worsening, defined as a >50% decrease in amplitude of the motor and/or sensory sum score.^18^

## Conclusion

Due to the unknown teratogenic potential of patisiran, the risk of neuropathy worsening associated with withholding treatment must of course be weighed against a potential teratogenic risk of treatment during pregnancy, pending further studies. Vitamin A levels need to be closely assessed, and substitution must be adapted accordingly, to avoid embryofetal adverse outcome due to VAD or toxicity. Women exposed to patisiran treatment at any point of the pregnancy can be enrolled in the currently ongoing exposure registry.

## Appendix

*Disease severity scores*: Polyneuropathy Disability Score (PND) stratifies patient disability into six stages: PND 0 no impairment; PND I sensory disturbances but preserved walking capacity; PND II impaired walking capacity but ability to walk without a stick or crutches; PND IIIa walking only with the help of one stick or crutch and IIIb with the help of two sticks or crutches and PND IV confined to a wheelchair or bedridden. Neuropathy Impairment Score is a composite score of clinical impairments (weakness, reflex loss, and sensory loss), range 0–180, with higher score indicating more impairment. Compound Autonomic Dysfunction Test is a questionnaire which evaluates the main symptoms of autonomic dysfunction, range 0–16 in females and 0–20 in males, with lower scores indicating more autonomic symptoms. Small fiber Neuropathy-Symptom Inventory Questionnaire is a questionnaire which evaluates somatic and autonomic symptoms related to small fiber neuropathy, range 0–45 for the follow-up, with higher score indicating more symptoms. Rasch-built Overall Disability Score is a disability score, range 0–48, with lower scores indicating more disability. Norfolk quality of life-diabetic neuropathy is a questionnaire of quality of life, range 4–136, with higher scores indicating worst quality of life.

*ESC*: For the measurement of ESC with Sudoscan, the lower limit of the norm was set at 60 μS for hands and 70 μS for feet.

*NCS motor and sensory sum scores*: The motor sum score is a composite of the amplitude of ulnar and fibular nerve compound muscle action potential in milli volt. The sensory sum score is a composite of the amplitude of ulnar (orthodromic testing) and sural (antidromic testing) nerve SNAP in microvolt.

## Declarations

**Ethics approval and consent to participate:** Not applicable.

**Consent for publication:** A written informed consent for publication was provided by the participant to the authors.

**Availability of data and materials:** The data that supports the findings of this study are available from the corresponding author upon reasonable request.
